# Dual-energy X-ray absorptiometry-assessed adipose tissues in metabolically unhealthy normal weight Asians

**DOI:** 10.1038/s41598-019-53557-9

**Published:** 2019-11-27

**Authors:** Yi-Chien Lu, Ying Chin Lin, Amy Ming-Fang Yen, Wing P. Chan

**Affiliations:** 1 Department of Radiology, Wan Fang Hospital, Taipei Medical University, Taipei, 116 Taiwan; 2 Department of Family Medicine, Wan Fang Hospital, Taipei Medical University, New Taipei City, 235 Taiwan; 3 Department of Family Medicine, School of Medicine, College of Medicine, Taipei Medical University, Taipei, 110 Taiwan; 4 School of Oral Hygiene, College of Oral Medicine, Taipei Medical University, Taipei, Taiwan; 5 Department of Radiology, School of Medicine, College of Medicine, Taipei Medical University, Taipei, 110 Taiwan

**Keywords:** Metabolic disorders, Risk factors

## Abstract

Normal body mass index (BMI) is associated with lower risk for cardiometabolic diseases. However, there is a subset of individuals with BMI in this range who present with this metabolic abnormality (called metabolically unhealthy normal weight, MUHNW). Here we aimed to assess the adipose characteristics of people with MUHNW using dual-energy X-ray absorptiometry (DXA). This study included 3259 people with normal BMI who underwent health examinations from January 1, 2007 through December 31, 2016. Body fat percentage (%BF), android-gynoid percent fat ratio (AG ratio), and visceral adipose tissue (VAT) were measured simultaneously using DXA CoreScan software. Those with MUHNW comprised 12.67% of the sample. Among those with MUHNW, 71.6% of the women and 56.5% of the men showed high VAT amounts, but less than 40% of either showed high %BFs. Furthermore, considering the combined effects of fat amount and distribution, a normal BMI accompanied by high AG ratio and/or high VAT mass but low %BF presents a much higher risk for metabolic syndrome than when %BF is high, most predominantly in women. In conclusion, using DXA-measured abdominal fat, particularly VAT accumulation, is clinically more important than using %BF when assessing metabolic syndrome in those with normal BMI.

## Introduction

Obesity, commonly defined using body mass index (BMI), has become a major noncommunicable disease risk factor around the world^1^. In 2016, more than 1.9 billion adults aged 18 years and older were overweight (BMI ≥ 25 kg/m^2^). Of these, over 650 million were obese (BMI ≥ 30 kg/m^2^). According to the latest data published by the World Health Organization, the prevalences of overweight and obesity among adults are 39% and 13%, respectively^2^. A high BMI can lead to metabolic syndrome (MetS), which is highly associated with type-2 diabetes and cardiovascular disease (CVD)^3–7^; however, subgroups of individuals do not have this phenotype^8,9^.

Increasing attention has been paid to two subgroups: those with metabolically unhealthy normal weight (MUHNW) and those who are metabolically healthy obese (MHO). In 2006, Meigs *et al*. studied 2902 Europeans without diabetes or CVD and found MetS in 7% of those with normal weight^10^. In another study, the National Health and Nutrition Examination Survey (NHANES) 1999–2004 indicated that 24% of normal-weight individuals have two or more metabolic abnormalities^11^. Similarly, a recent large cross-sectional study showed that 8.1% of 11,884 normal weight Chinese had MUHNW. It also showed that the prevalence of MUHNW in women tended to be higher than in men^12^.

Although BMI is the most commonly anthropometric index used to define obesity, its ability to accurately predict body fat content and distribution is limited. The body fat percentages (%BFs) are wide ranging among women (4.6–51.1%) and men (5.6–31.2%) with normal BMIs^13^. Reportedly, individuals with normal BMIs but high %BFs are at increased risk of cardiometabolic disease and CV mortality^14,15^. Additionally, risk of metabolic abnormality is nearly 3-fold higher in those with high %BFs than those with low %BFs^16^. People with normal BMIs showed that higher %BF was associated with higher prevalence of high blood pressure, hyperglycemia, and dyslipidemia^17^.

Regional fat distribution is thought to be more closely associated than total body fat amount with MetS^18,19^. Those with excess abdominal (android) fat deposition, especially visceral adipose tissue (VAT) accumulation, are at higher risk of presenting insulin resistance and MetS^20–22^. Several studies suggest that the android-gynoid percent fat ratio (AG ratio) is an important determinant of metabolic disease risk^23–25^. Furthermore, VAT has a greater pathogenic effect than subcutaneous adipose tissue (SAT)^18,19,22,26^.

Most studies have used only anthropometric measurements, such as waist circumference (WC) or waist-hip ratio (WHR) to assess abdominal obesity^27–29^. Although several studies used computed tomography (CT) to measure VAT, its widespread application is limited by high costs and considerable radiation exposure. Compared to CT, dual-energy X-ray absorptiometry (DXA) is less expensive and exposes patients to negligible radiation in that it uses attenuation of high- and low-energy X-rays passing through the body to distinguish between bone, lean tissue, and fatty tissue^30^. Besides measuring body composition, the automated software, CoreScan, can further estimate VAT within the android region, and the value using DXA is significantly correlated with that using CT^31^.

We therefore aimed to evaluate the use of several fat-related measurements (including %BF, AG ratio, and VAT and SAT masses), assessed using DXA, to identify the risk of MUHNW in a large cohort. In addition, we further characterized fat distribution in those with MUHNW and various %BFs.

## Results

The cross-sectional analyses included 3259 individuals (1904 women; Table 1). The %BF, android fat mass, AG ratio, VAT, and the ratio of VAT mass to SAT mass (VS ratio) differ significantly between metabolically healthy normal weight (MHNW) people and MUHNW people in both sexes. However, gynoid fat mass and SAT were significantly greater in MUHNW people only among the women. Overall, men had a higher prevalence of hypertension, high glucose, high triglycerides, and low HDL. However, more of the women (26.42%) had central obesity than men (5.54%). The prevalence of MUHNW was 12.03% for women and 13.58%, for men.

**Table 1:** Characteristics of the study participants^a^.

	Women			Men		
	Total	MHNW	MUHNW	Total	MHNW	MUHNW
No.	1904	1675	229	1355	1171	184
Age (y)	47.38 ± 11.04	46.04 ± 10.44	57.18 ± 10.32^§^	50.43 ± 11.40	49.91 ± 11.41	53.76 ± 10.78^§^
Height (cm)	158.56 ± 5.56	158.73 ± 5.39	157.27 ± 6.56^#^	170.14 ± 6.17	170.20 ± 6.16	169.77 ± 6.26
Weight (kg)	53.65 ± 4.85	53.45 ± 4.77	55.14 ± 5.17^§^	64.23 ± 6.00	64.02 ± 6.00	65.61 ± 5.87^#^
BMI (kg/m^2^)	21.33 ± 1.44	21.20 ± 1.42	22.27 ± 1.20^§^	22.16 ± 1.32	22.07 ± 1.35	22.72 ± 0.94^§^
%BF	32.80 ± 4.59	32.59 ± 4.59	34.40 ± 4.28^§^	21.93 ± 4.79	21.58 ± 4.81	24.15 ± 4.08^§^
Android fat mass (g)	1.46 ± 0.39	1.41 ± 0.37	1.80 ± 0.34^§^	1.55 ± 0.46	1.50 ± 0.45	1.85 ± 0.40^§^
Gynoid fat mass (g)	3.56 ± 0.67	3.59 ± 0.66	3.37 ± 0.69^§^	2.43 ± 0.58	2.42 ± 0.58	2.49 ± 0.58
AG ratio	0.96 ± 0.15	0.94 ± 0.14	1.11 ± 0.14^§^	1.33 ± 0.23	1.31 ± 0.23	1.45 ± 0.21^§^
SAT (g)	1038.03 ± 266.15	1028.95 ± 266.18	1104.39 ± 256.87^§^	775.89 ± 272.11	773.06 ± 274.45	793.92 ± 256.69
VAT (g)	420.20 ± 229.33	383.08 ± 200.10	691.70 ± 246.58^§^	772.70 ± 355.14	728.02 ± 333.25N	1057.04 ± 359.25^§^
VS ratio	0.42 ± 0.25	0.39 ± 0.22	0.67 ± 0.31^§^	1.19 ± 1.55	1.10 ± 0.84	1.74 ± 3.60*
Waist (cm)	75.74 ± 6.31	74.93 ± 5.93	81.67 ± 5.81^§^	81.30 ± 5.32	80.73 ± 5.20	84.96 ± 4.60^§^
SBP (mmHg)	111.48 ± 17.27	109.50 ± 16.34	125.93 ± 17.01^§^	120.18 ± 16.72	118.44 ± 15.84	131.24 ± 17.94^§^
DBP (mmHg)	72.01 ± 10.49	71.10 ± 9.98	78.73 ± 11.68^§^	76.94 ± 10.72	75.97 ± 10.16	83.09 ± 12.07^§^
FG (mg/dl)	93.97 ± 15.17	91.70 ± 9.85	110.58 ± 29.89^§^	99.30 ± 22.48	96.59 ± 18.79	116.54 ± 33.68^§^
Triglycerides (mg/dl)	85.21 ± 50.30	75.60 ± 35.16	155.51 ± 80.01^§^	115.36 ± 83.54	100.99 ± 49.65	206.79 ± 161.71^§^
HDL-C (mg/dl)	58.48 ± 14.32	60.02 ± 13.93	47.22 ± 11.88^§^	46.39 ± 12.50	47.72 ± 12.46	37.91 ± 8.93^§^
WBC count (10^3^/μl)	5.49 ± 1.46	5.44 ± 1.44	5.82 ± 1.53^§^	5.98 ± 1.67	5.86 ± 1.61	6.71 ± 1.85^§^
Central obesity^b^	26.42%	20.4%	70.3%	5.54%	3.1%	21.2%
Hypertension^c^	20.01%	14.3%	61.6%	35.28%	29.8%	70.1%
High glucose^d^	21.06%	14.4%	69.9%	34.69%	27.2%	82.1%
High TG^e^	9.61%	3.3%	55.9%	21.33%	13.4%	71.7%
Low HDL^f^	28.57%	22.6%	72.1%	30.85%	23.6%	77.2%
MetS	12.03%	−	−	13.58%	−	—

MHNW, metabolically healthy normal weight; MUHNW, metabolically unhealthy normal weight; BMI, body mass index; %BF, total body fat percentage; AG ratio, ratio of android to gynoid %fat; SAT, subcutaneous adipose tissue; VAT, visceral adipose tissue; VS ratio, ratio of VAT to SAT; SBP, systolic blood pressure; DSP, diastolic blood pressure; FG, fasting glucose; HDL, high-density lipoprotein cholesterol; WBC, white blood cell; MetS, metabolic syndrome.

*p < 0.05, ^#^p < 0.01, ^§^p < 0.001 in the *t-test* between MHNW and MUHNW.

^a^Data are presented as mean ± SD.

^b^Waist circumference ≥ 80 cm for women and ≥90 cm for men.

^c^SBP ≥ 130 mm Hg or DBP ≥ 85 mm Hg.

^d^Fasting glucose ≥ 100 mg/dl.

^e^Fasting serum triglycerides ≥ 150 mg/dl.

^f^HDL < 50 mg/dl for women and <40 mg/dl for men.

### Risk of having the MUHNW phenotype

**Figure 1:**
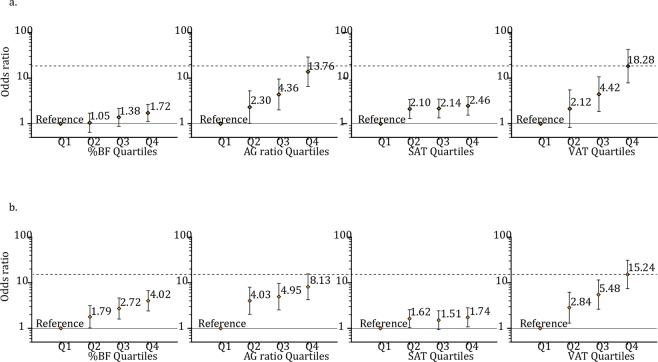
Odds ratios for metabolically unhealthy normal weight people grouped into quartiles for various fat indicators, independently in (**a**) women and (**b**) men. Quartile points for %BF were 29.84, 33.10, and 36.10 in women and 19.18, 22.27, and 25.09 in men; for AG ratio, they were 0.86, 0.96, and 1.05 in women and 1.19, 1.32, and 1.47 in men; for SAT mass, they were 863 g, 1024 g, and 1195 g in women and 602 g, 782 g, and 949 g in men; and for VAT mass, they were 250 g, 391 g, and 553 g in women and 513 g, 752 g, and 1005 g in men. %BF, total body fat percentage; AG ratio, android to gynoid percent fat ratio; SAT, subcutaneous adipose tissue; VAT, visceral adipose tissue. Adjusted odds ratios (95% CI) were analyzed using logistic regression with age as a covariate.

In both sexes, after adjusting for age, the odds for MUHNW were statistically increased with an increase in any fat indicator. The ORs for MetS, by comparing Q4 to Q1 for %BFs, AG ratios, and SAT and VAT masses, were 1.72 (95% CI, 1.11–2.65), 13.76 (6.57–28.84), 2.46 (1.54–3.93), and 18.28 (7.84–42.64), respectively, for women (Fig. 1a) and 4.02 (2.41–6.72), 8.13 (4.23–15.62), 1.74 (1.08–2.82), and 15.24 (7.43–31.29), respectively, for men (Fig. 1b). In both sexes, increasing prevalence of the MUHNW phenotype was associated with increasing %BF, AG ratio, and VAT mass. The prevalence was lowest in Q1_VAT and highest in Q4_VAT (in the Supplementary Table S1).

### Dependence of phenotype and risk of MUHNW

**Figure 2:**
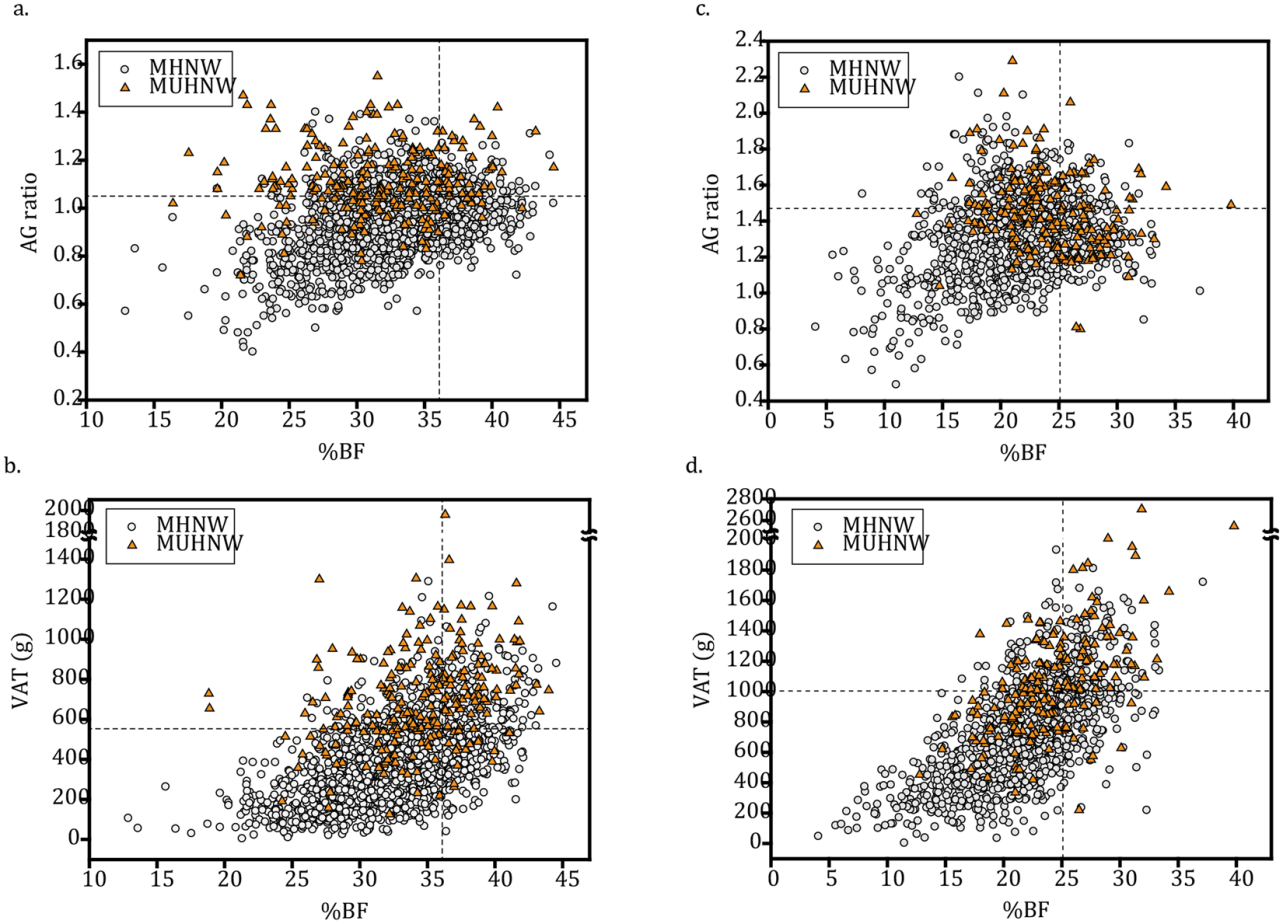
Scatter plots of percent of total body fat versus AG ratio and/or VAT mass. (**a**) and (**b**) are scatter plots for %BF versus AG ratio and/or VAT mass in women, respectively. (**c**) and (**d**) are scatter plots for %BF versus AG ratio and/or VAT mass in men, respectively. The vertical lines indicate the sex-specific 4th quartile point for %BF (36.10 in women and 25.09 in men), and the horizontal lines indicate the sex-specific 4th quartile point for AG ratio (1.05 in women and 1.47 in men) or for VAT mass (553 g in women and 1005 g in men). %BF, total body fat percentage; AG ratio, android to gynoid percent fat ratio; VAT, visceral adipose tissue.

Of 229 women with MUHNW, 68.1% showed high (in Q4 range) AG ratios, 71.6% showed high VAT masses, and only 38.0% showed high %BF (Fig. 2a,b). Of 184 men with MUHNW, these values were 44.6%, 56.5%, and 40.2% (Fig. 2c,d). In contrast, no matter the sex, less than 25% of those MHNWs showed high %BFs, AG ratios, or VAT masses (Table 2).

**Table 2:** Adiposity features in those with MHNW and MUHNW.

		Women				Men			
		Q1	Q2	Q3	Q4	Q1	Q2	Q3	Q4
%BF	MHNW	438 (26.1%)	434 (25.9%)	412 (24.6%)	391 (23.3%)	317 (27.1%)	303 (25.9%)	285 (24.3%)	266 (22.7%)
	MUHNW	37 (16.2%)	43 (18.8%)	62 (27.1%)	87 (38.0%)	21 (11.4%)	36 (19.6%)	53 (28.8%)	74 (40.2%)
AG ratio	MHNW	469 (28.0%)	471 (28.1%)	399 (23.8%)	336 (20.1%)	322 (27.5%)	293 (25.0%)	289 (24.7%)	267 (22.8%)
	MUHNW	8 (3.5%)	23 (10.0%)	42 (18.3%)	156 (68.1%)	11 (6.0%)	41 (22.3%)	50 (27.2%)	82 (44.6%)
VAT	MHNW	470 (28.1%)	458 (27.3%)	433 (25.9%)	314 (18.7%)	328 (28.0%)	314 (26.8%)	294 (25.1%)	235 (20.1%)
	MUHNW	6 (2.6%)	17 (7.4%)	42 (18.3%)	164 (71.6%)	9 (4.9%)	25 (13.6%)	46 (25.0%)	104 (56.5%)

MHNW, metabolically healthy normal weight; MUHNW, metabolically unhealthy normal weight; %BF, total body fat percentage; AG ratio, ratio of android to gynoid %fat; VAT, visceral adipose tissue.

Quartile points for %BF were 29.84, 33.10, and 36.10 in women and 19.18, 22.27, and 25.09 in men; for AG ratio, they were 0.86, 0.96, and 1.05 in women and 1.19, 1.32, and 1.47 in men, and for VAT mass, they were 250 g, 391 g, and 553 g in women and 513 g, 752 g, and 1005 g in men.

Data are presented as the number of participants (percentage).

**Figure 3:**
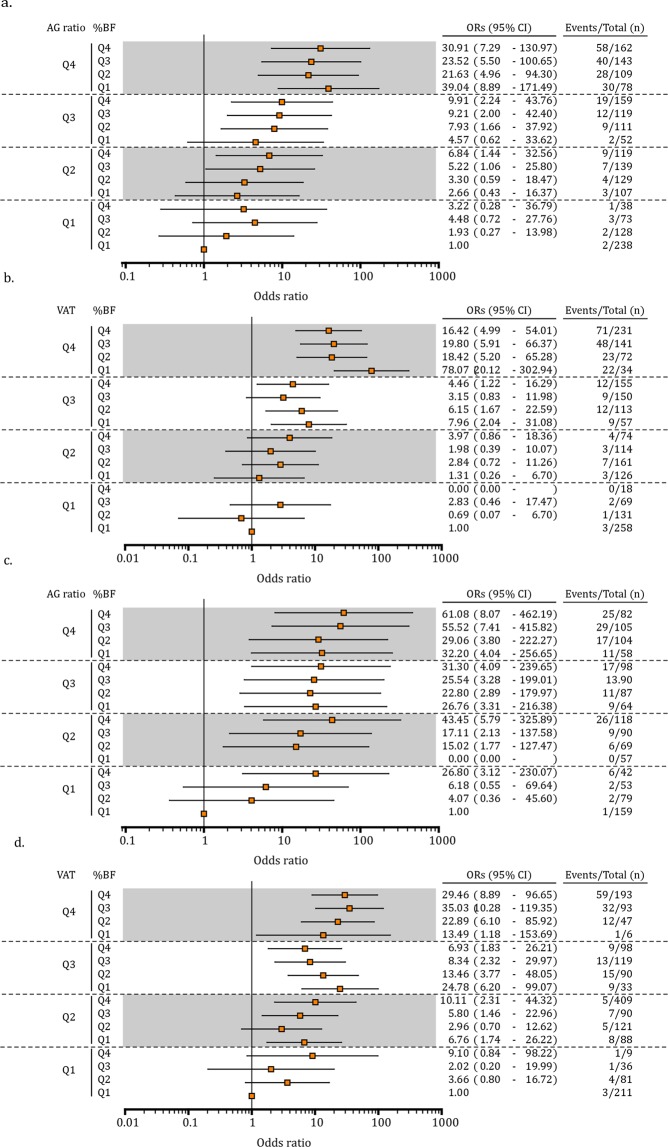
Risk of metabolically unhealthy normal weight according to %BF versus AG ratio and/or VAT quartiles in (**a,b**) women and (**c,d**) men. Quartile points for %BF were 29.84, 33.10, and 36.10 in women and 19.18, 22.27, and 25.09 in men, for AG ratio, they were 0.86, 0.96, and 1.05 in women and 1.19, 1.32, and 1.47 in men, and for VAT mass, they were 250 g, 391 g, and 553 g in women and 513 g, 752 g, and 1005 g in men. %BF, total body fat percentage; AG ratio, android to gynoid percent fat ratio; VAT, visceral adipose tissue. Adjusted odds ratios (95% CI) were analyzed using logistic regression with age as a covariate.

We stratified participants into %BF/AG ratio and %BF/VAT mass quartile groups (Fig. 3) and found that the risk of MetS was much higher if the AG ratio and/or VAT mass were high (Q4) despite low %BF (Q1). In women, using Q1_AG ratio/Q1_%BF as the reference group, the risk of MetS was increased more than 20-fold for those in the Q4_AG ratio group (Fig. 3a). The ORs for MetS were highest in the Q4_AG ratio/Q1_%BF group (39.04 [8.89–171.49]), followed by the Q4_AG ratio/Q4_%BF group (30.91 [7.29–130.97]) and the Q1_AG ratio/Q4_%BF group (3.22 [0.28–36.79]). In addition, using Q1_VAT/Q1_%BF as the reference group, the odds of MetS increased more than 15-fold in Q4_VAT women (Fig. 3b), and importantly, over 70-fold in Q4_VAT/Q1_%BF women (78.07 [20.12–302.94]).

In men, the risk of MetS was increased more than 30-fold for most of those in the Q4_ AG ratio group compared with the Q1_AG ratio/Q1_%BF group (Fig. 3c) and was the highest in the Q4_AG ratio/Q4_%BF group (61.08 [8.07–462.19]). The risk was higher in Q4_AG ratio/Q1_%BF men (32.20 [4.04–256.63]) than in Q1_AG ratio/Q4_%BF men (26.80 [3.12–230.27]). Moreover, the risk was more than 10-fold higher in Q4_VAT/Q1_%BF men (from 13.49 to 35.03) than in Q1_VAT/Q1_%BF men (Fig. 3d).

### Relative risk of MetS attributable to %BF, AG ratio, and VAT mass

Analyses using each fat indicator alone (Table 3) revealed that ORs ranged from 1.22 to 3.02 in women and from 1.17 to 2.43 in men (Model 1, all P < 0.05). Using %BF, AG ratio, and SAT and VAT masses as the covariates, multiple regression analysis showed that an increasing AG ratio and VAT mass significantly increased the odds for MetS per quartile in both sexes. However, increasing %BF decreased the risk of MetS per quartile in women (0.78 [0.64–0.95]) but not in men (1.01 [0.79–1.28]). The SAT mass had no significant effect in either (Model 2). Correlations between %BF, AG ratio, and SAT and VAT masses are shown in the Supplementary Table S2.

**Table 3:** Increase in relative risk of MetS attributable to %BF, AG ratio, and VAT, alone or in combination, when BMI was normal^a^.

Covariate^b^	Women		Men	
	Odds ratio (95% CI)	P-value	Odds ratio (95% CI)	P-value
**Model 1: independent fat indicators**				
%BF	1.22 (1.06–1.40)	0.01	1.56 (1.34–1.82)	<0.001
AG ratio	2.50 (2.07–3.00)	<0.001	1.73 (1.48–2.02)	<0.001
SAT	1.27 (1.11–1.46)	0.001	1.17 (1.01–1.35)	0.04
VAT	3.02 (2.44–3.72)	<0.001	2.43 (2.02–2.92)	<0.001
**Model 2: multivariate model with %BF, AG ratio, SAT, and VAT as model covariates**				
%BF	0.78 (0.64–0.95)	0.02	1.01 (0.79–1.28)	0.97
AG ratio	1.65 (1.32–2.06)	<0.001	1.26 (1.04–1.53)	0.02
SAT	1.13 (0.94–1.36)	0.21	1.05 (0.87–1.28)	0.61
VAT	2.59 (2.00–3.36)	<0.001	2.16 (1.66–2.82)	<0.001

^a^As a per-quartile increase.

^b^All models were adjusted for age.

## Discussion

We made three key findings in our sample with normal BMIs: (1) 12.67% of those with normal weight had MUHNW, (2) all fat variables (%BF, AG ratio, SAT mass, and VAT mass) increased the risk for MetS, and (3) total fat amount and distribution were simultaneously assessed in those with MUHNWs. We distinguished between the MHNW and MUHNW adiposity phenotypes and showed that the risk for MetS was highest in those with normal BMIs and with high AG ratios or/and high VAT masses but low %BFs.

Because a metabolically healthy status has not been consistently defined, the prevalence of MUHNW has varied from 7% to 40%^9,10,32,33^. In this study, we defined MetS using APT III criteria and estimated the prevalence of MUHNW in Taiwan to be 12.67%. Similar estimates have been reported for Korea (12.7%)^34^, with Chinese (8.1%)^12^ and Europeans (7.1%)^10^, notably lower. Using two or more metabolic abnormalities (excluding abdominal obesity) to define MUHNW, Ogorodnikova *et al*. analyzed 17,544 participants, finding that 30.5% of women and 39.8% of men had MUHNW, the prevalence being greater in African Americans than in White people^33^. These results indicate that a certain proportion of those with normal BMIs had adverse metabolic features regardless of ethnic group.

The associations between higher BMI and increasing CVD and mortality have been recently questioned^35,36^. Increasing attention has focused on the features of obese individuals with benign metabolic status and those of normal weight with adverse metabolic status^37–40^. In a recent systematic review, people with MUHNWs had higher risk of all-cause mortality (relative risk, 3.14) than those who were MHO (1.19) and those with metabolically unhealthy obesity (2.65)^41^. Several studies also demonstrated worse lipid profiles and poorer insulin sensitivity in those with MUHNW than those who were MHO^11,37,42^. Thus, maintaining BMI within the normal range is no longer the only indicator of health.

Recent studies suggest that body fat amount would be a better indicator of obesity than BMI^13,43,44^. Although BMI is the most popular and widely used method to assess obesity, it does not truly reflect body fat mass and its distribution. Kennedy *et al*. found that %BF varies widely in each BMI category in both sexes. Furthermore, over one-third of study participants are misclassified using BMI rather than DXA %BF^13^. Evidence shows that individuals with normal BMIs but excessive %BFs (a condition referred to as normal weight obesity [NWO]) tend to develop several metabolic diseases^45,46^. In our study, high %BF was associated with increasing risk of MetS despite normal BMI. This result was buttressed by Zhu’s study showing an association between %BF range and MetS^47^ and others, who showed associations between NWO and cardiometabolic dysregulation^14–16^. Analyzing 6171 participants of the NHANES III, MetS, hypertension, and dyslipidemia were more prevalent in those with NWO than without it^14^. By contrast, Ortega *et al*. reported that BMI is a better predictor of CVD mortality than total body fat measurement^4^. Failure to consider regional fat distribution might account for this inconsistency.

Numerous studies found that abdominal or truncal obesity increases metabolic and CVD risk in both children and adults^25,48,49^. Even with normal BMIs, those with central obesity based on either WC or WHR have greater risk for CV mortality^27–29^. Sharma *et al*. studied 7057 coronary artery disease patients older than 65 years, finding those with normal BMIs and high WCs as well as those with high WHRs to have the highest mortality risks^28^. Using 5100 Mexican adults, another study showed that WC is a more accurate detector of metabolic disorders than %BF^50^. Using our more precise technique, DXA, to evaluate android and gynoid fat masses, we found high AG ratios in 57.6% of those with MUHNW (68.1% of women and 44.6% of men), but high %BFs in less than 40% of the sample. Similarly, Fu indicated a stronger association between AG ratio, compared to %BF, and metabolic risk in those with normal BMIs^49^. Other studies have also demonstrated correlations between increasing AG ratio and either higher triglycerides or lower HDL^24,49^. In addition, Walton *et al*. found that body fat distribution (as determined by DXA in 103 men) rather than fat amount was related to adverse lipid profiles^51^. These results are consistent with our observation and support the suggestion that excess abdominal fat accumulation results in adverse metabolic status.

Abdominal fat accumulates in two adipose tissues–VAT and SAT. Using DXA-derived CoreScan software, we observed that two-thirds of our sample with MUHNW showed high VAT masses. Furthermore, those with high VAT masses had greater MetS risk than those with high %BFs. A vast amount of evidence supports the notion that VAT is a pathogenic fat deposit and has adverse metabolic consequences including predisposition to hypertension, insulin resistance, diabetes, and MetS^19,20,26,52^. In Japan, a 2017 study demonstrated a dose-dependent relationship between VAT mass and metabolic risk factors among people with normal or higher BMIs^22^. However, a longitudinal study of elderly participants reported by Kang *et al*. indicated a greater association between android fat deposits and MetS compared to VAT mass^53^, contradicting our observation that VAT, not SAT mass, is more closely associated with MetS in those with normal BMIs. This discordance might be explained by a difference in methodology for measuring android fat deposits and VAT. In Kang’s study, the former (represented by fat mass in the upper abdomen) was measured using DXA, but VAT was measured using a single CT slice at the umbilicus level. This assumption is supported by a recent report suggesting a stronger correlation between insulin resistance and fat amount when estimated at multiple L1-L5 levels rather than a single L3 level^54^.

DXA-dedicated CoreScan software has been newly developed to estimate VAT within the android region^55^. Using DXA to measure VAT is as accurate as using CT^31,56^. Although CT is considered the gold standard, it is expensive and exposes the patient to considerable radiation. Measurements of WC and WHR to estimate VAT do not accurately reflect VAT mass. In large clinical studies, DXA offers lower radiation exposure, lower cost, and easier determination of total and regional body composition^57^.

Our results suggest that NWO differs from MUHNW. Substantial evidence indicates that individuals with NWO, defined either by high %BF or by central obesity, have greater risk for MetS than others; however, only a subset of those with NWO suffer from metabolic dysfunctions. Using the DXA-derived CoreScan tool to assess ~3000 individuals with normal BMIs, we further proved the concept that excess VAT accumulation was a better predictor than body fat amount. The high VAT mass/low %BF group, in particular, had an extremely high risk of MetS, exceeding that for those with high VAT mass/high %BF. Accumulated VAT is a major contributor to risks of CVD and MetS^58,59^. It has a stronger association with these factors than does SAT^26,60^. In this study of individuals with the same %BF, MetS risk increased with greater VAT mass. On the other hand, in individuals with the same VAT mass, the risk of MetS was greater when %BF was low rather than high. This indicates that %BF had limited utility for assessing MetS risk in those with normal BMIs.

This study’s strengths are: (1) it had the largest sample size and collection of VAT data derived using DXA CoreScan in an Asian ethnic group, (2) it used simultaneously collected total fat amounts and distributions in Asians with MUHNW, and (3) it included only people with normal BMIs to avoid body size bias when measuring %BF with DXA^61^. A weakness of this hospital-based study was its limited population diversity. In addition, because of its retrospective nature, no data on factors, such as WHR, insulin sensitivity, and physical activity, reportedly associated with MetS, were obtained.

In conclusion, this work draws attention to the risk of metabolic diseases in those without obvious risks for obesity. Our findings identified excess abdominal visceral fat accumulation as a major characteristic of MUHNW. Notably, a normal BMI accompanied by high AG ratio and/or high VAT mass but low %BF presents a much higher risk for MetS than when %BF is high, most prominently in women. These findings not only support the notion that body fat distribution is more impactful than body fat amount, but they further draw attention to the idea of visceral obesity with normal BMI. DXA-measured abdominal VAT accumulation is more clinically important than %BF when assessing MetS risk in those with normal BMIs.

## Patients and Methods

### Study design and participants

This cross-sectional study analyzed the medical records of 6925 patients who received annual health examinations and full-body DXA scans from a single medical center in Taiwan between January 1, 2007 and December 31, 2016. Inclusion criteria were: (1) aged 20 years or more, (2) body composition determined by DXA scan, (3) normal BMI normal (18.5–24.0 kg/m^2^), and (4) Chinese/Taiwanese nationality. The first medical record for each patient was used. Follow-up examinations and incomplete records (missing anthropometric, biochemical, or body composition data) were excluded. Finally, 3259 participants were included. This study was performed in accordance with relevant guidelines and was conducted after approval by the Taipei Medical University-Joint Institutional Review Board (Number: N201712053). Informed consent was waived because of its retrospective nature.

### Anthropometric and biochemical measurements

Body weight, precise to 0.1 kg, was determined using an electronic scale; height, to 0.1 cm, was determined using a fixed stadiometer; waist circumference (WC) was determined using a measuring tape at the midpoint between the lowest rib and iliac crest in the standing position; and blood pressure was determined using a standard digital sphygmomanometer while the participant was seated. The BMI was calculated as weight in kilograms divided by height in meters, squared (kg/m^2^). All blood samples were obtained after fasting for 8 h.

### Body composition measurements

Whole-body composition was measured using the DXA scan (Lunar Prodigy, version 9.1; GE Healthcare, Madison, WI); all measurements were performed by three experienced technicians certified by the International Society for Clinical Densitometry, and all protocols and procedures were strictly followed. Total %BF was defined as the ratio between total fat mass and total body mass. The boundaries of the regions of interest (ROIs) for determining regional body composition were defined by the software manufacturer: (a) the android ROI was defined by the pelvis cut line (lower boundary), above the pelvis cut line by 20% of the distance between the pelvis and neck cut lines (upper boundary), and arm cut lines (lateral boundaries), and (b) the gynoid ROI was below the pelvis cut line by 1.5 times the height of the android ROI (lower boundary), above the lower boundary by twice the height of the android ROI (upper boundary), and the outer leg cut lines (lateral boundaries). The AG ratio was defined as the ratio between the percent fat in the android (central) ROI and that in the gynoid (hip and thigh) ROI. The CoreScan software was also used to estimate the VAT mass within the android ROI. The SAT mass was defined as the android region fat mass minus the VAT mass. The VS ratio was defined as the ratio between VAT mass and SAT mass.

### Definition of metabolically unhealthy normal weight

Metabolically unhealthy normal weight was defined as having normal BMI (18.5–24.0 kg/m^2^) while having MetS, which was defined as meeting at least 3 criteria defined by the National Cholesterol Education Adult Treatment Panel III (ATP III)^62^: (a) WC at least 90 cm in men and 80 cm in women; (b) systolic blood pressure (SBP) of at least 130 mm Hg and/or diastolic blood pressure (DSP) of at least 85 mm Hg or taking medication for hypertension; (c) fasting glucose of at least 100 mg/dl or on a drug treatment for diabetes; (d) high-density lipoprotein (HDL) cholesterol lower than 40 mg/dl for men and 50 mg/dl for women; and (e) triglycerides of at least 150 mg/dl or on a drug treatment for hyperlipidemia.

### Statistical analysis

The database was established using Excel and SPSS software. Sample characteristics are summarized using the median (range 25th–75th percentile). An independent Mann-Whitney test was used to compare men with women based on each measurement. The sample was divided into sex-specific quartiles based on %BF, AG ratio, and SAT and VAT masses, allowing exact comparisons between these variables. The McNemar chi-square test was used for evaluating differences in MUHNW prevalence. Binary logistic regression models were formed to compute the odds ratios (ORs) of having the MUHNW phenotype. The sample was further stratified according to sex-specific quartiles into sixteen groups so that the effects of fat distribution on the MUHNW phenotype could be clarified. The ORs and 95% confidence intervals (CIs) were estimated separately for each group using Q1_%BF/Q1_AG ratio or Q1_%BF/Q1_VAT as a reference. Age was accounted for in all analyses. Statistical analyses were performed using PASW Statistics version 18.0 (SPSS Inc., Chicago, IL), and when P < 0.05, a statistically significant difference was recognized.

## Supplementary information

SUPPLEMENTAL ONLINE MATERIALS

## Data Availability

The datasets generated during and/or analyzed during the current study are available from the corresponding author on reasonable request.
