# Supplementary material for: Dual-energy X-ray absorptiometry-assessed adipose tissues in metabolically unhealthy normal weight Asians
Source: Sci Rep. 2019 Nov 27;9:17698. doi: 10.1038/s41598-019-53557-9 (PMC6881341; doi:10.1038/s41598-019-53557-9)
Supplement: Supplementary file 1 — SUPPLEMENTAL ONLINE MATERIALS [file 41598_2019_53557_MOESM1_ESM.docx]

Dual-energy X-ray absorptiometry-assessed adipose tissues in metabolically unhealthy normal weight Asians

Yi-Chien Lu, PhD^1,Δ^, Ying Chin Lin, MD^2,3,Δ^, Amy Ming-Fang Yen, PhD^4^, Wing P. Chan, MD^1, 5,*^

1. Department of Radiology, Wan Fang Hospital, Taipei Medical University, Taipei 116, Taiwan, Republic of China;
2. Department of Family Medicine, Shuang Ho Hospital, Taipei Medical University, New Taipei City 235, Taiwan, Republic of China;
3. Department of Family Medicine, School of Medicine, College of Medicine, Taipei Medical University, Taipei 110, Taiwan, Republic of China;
4. School of Oral Hygiene, College of Oral Medicine, Taipei Medical University, Taipei, Taiwan, Republic of China;
5. Department of Radiology, School of Medicine, College of Medicine, Taipei Medical University, Taipei 110, Taiwan, Republic of China

SUPPLEMENTAL ONLINE MATERIALS

**Table S1. The prevalence of metabolically unhealthy normal weight according to fat indicators**

|  | **Q1** | **Q2** | **Q3** | **Q4** | **P-value** |
| --- | --- | --- | --- | --- | --- |
| ***Women (n=1904)*** |  |  |  |  |  |
| %BF | 37 (7.8%) | 43 (9.0%) | 62 (13.1%) | 87 (18.2%) | <.001 |
| AG ratio | 8 (1.7%) | 23 (4.7%) | 42 (9.5%) | 156 (31.7%) | <.001 |
| SAT | 32 (6.8%) | 61 (12.7%) | 64 (13.4%) | 72 (15.1%) | .001 |
| VAT | 6 (1.3%) | 17 (3.6%) | 42 (8.8%) | 164 (34.3%) | <.001 |
| ***Men (n=1355)*** |  |  |  |  |  |
| %BF | 21 (6.2%) | 36 (10.6%) | 53 (15.7%) | 74 (21.8%) | <.001 |
| AG ratio | 11 (3.3%) | 41 (12.3%) | 50 (14.7%) | 82 (23.5%) | <.001 |
| SAT | 38 (11.2%) | 54 (15.9%) | 45 (13.3%) | 47 (13.8%) | .36 |
| VAT | 9 (2.7%) | 25 (7.4%) | 46 (13.5%) | 104 (30.7%) | <.001 |

%BF, total body fat percentage; AG ratio, android to gynoid %fat ratio; SAT, subcutaneous adipose tissue; VAT, visceral adipose tissue.

Quartile points for %BF were 29.84, 33.10, and 36.10 in women and 19.18, 22.27, and 25.09 in men; for AG ratio, they were 0.86, 0.96, and 1.05 in women and 1.19, 1.32, and 1.47 in men; for SAT mass, they were 863 g, 1024 g, and 1195 g in women and 602 g, 782 g, and 949 g in men; and for VAT mass, they were 250 g, 391 g, and 553 g in women and 513 g, 752 g, and 1005 g in men.

Data were presented as the MUHNW numbers (percentage).

P value for difference among the Q1 to Q4.

**Table S2. Correlations among various fat measurements^a,b^**

|  | %BF | AG ratio | SAT | VAT |
| --- | --- | --- | --- | --- |
| %BF | 1.00 | 0.36 | 0.68 | 0.52 |
| AG ratio | 0.26 | 1.00 | 0.47 | 0.69 |
| SAT | 0.60 | 0.16 | 1.00 | 0.24 |
| VAT | 0.66 | 0.57 | 0.06 | 1.00 |

^a^All P-values <.05.

^b^Gray shading represents correlations in men; no shading represents correlations in women.
